# Antitumor activity of nivolumab on hemodialysis after renal allograft rejection

**DOI:** 10.1186/s40425-016-0171-8

**Published:** 2016-10-18

**Authors:** Michael Ong, Andrea Marie Ibrahim, Samuel Bourassa-Blanchette, Christina Canil, Todd Fairhead, Greg Knoll

**Affiliations:** 1 Department of Medicine, The Ottawa Hospital, 501 Smyth Road, Ottawa, ON K1H 8L6 Canada; 2 The Ottawa Hospital Research Institute, 501 Smyth Road, Ottawa, ON K1H 8L6 Canada

**Keywords:** Melanoma, Acute allograft rejection, Organ transplant, Hemodialysis, Anti-PD-1 therapy, Nivolumab

## Abstract

**Background:**

Nivolumab (Opdivo™) is a novel IgG4 subclass programmed death-1 (PD-1) inhibiting antibody that has demonstrated breakthrough-designation anti-tumor activity. To date, clinical trials of nivolumab and other checkpoint inhibitors have generally excluded patients with solid organ transplantation and patients with concurrent immunosuppression. However, organ transplant recipients are at high-risk of development of malignancy as a result of suppressed immune surveillance of cancer.

**Case presentation:**

We illustrate the outcomes of a 63 year-old type I diabetic female patient who developed pulmonary metastatic, *BRAF* wild-type cutaneous melanoma 10 years after renal transplantation. After downward titration of the patient’s immunosuppressive medications and extensive multidisciplinary review, she was treated with nivolumab in the first-line setting. Within 1 week of administration, the patient experienced acute renal allograft rejection, renal failure and concurrent diabetic ketoacidosis due to steroid therapy. Allograft function did not return, but patient made a full clinical recovery after being placed on hemodialysis. Subsequently, the patient had clinical disease progression off therapy and required re-challenge with nivolumab on hemodialysis, resulting in ongoing clinical and radiographic response.

**Conclusions:**

This case illustrates multiple practical challenges and dangers of administering anti-PD1 immune checkpoint inhibitors to patients with solid-organ transplantation including need for titration of immunosuppressive medications, risks of allograft rejection, and treatment during hemodialysis.

## Background

Novel cancer immunotherapies targeting programmed-death-1 (PD-1) and its ligand (PD-L1) have demonstrated remarkable anti-cancer activity and survival benefit leading to regulatory approvals in metastatic melanoma, non-small cell lung cancer, and renal cell carcinoma [1, 2]. Clinical trials of nivolumab (Opdivo™), an IgG4 subclass PD-1-inhibiting antibody, and other similar immune checkpoint inhibitors have generally excluded patients with solid organ transplantation and patients with concurrent immunosuppression. However, organ transplant recipients are a high-risk cohort for developing metastatic cancer as a result of suppressed immune surveillance [3]. Immune checkpoint inhibition with anti-cytotoxic T-lymphocyte-associated protein-4 (CTLA-4) antibodies have previously been reported as successfully and safely administered with low dose-immunosuppression in liver transplant [4, 5] and renal transplant patients [6]. Recent literature has seen an emerging trend of anti-PD-1 medications being linked to rejection in transplant patients [7–10]. Herein we report a case of acute renal allograft rejection seven days after administration of first dose of nivolumab in the setting of concomitant radiological response of the metastatic melanoma.

## Case presentation

A 63 year-old female with longstanding type-1 diabetes mellitus and hypertension developed chronic renal failure in 2002 and underwent a pre-emptive renal allograft transplant from her donor husband in 2004 without requiring prior dialysis. Both the donor and recipient were cytomegalovirus-negative. She received basiliximab 20 mg prior to her transplant surgery and another 20 mg on day 4 post-transplant. Following transplantation she was immunosuppressed with mycophenolate mofetil, prednisone and tacrolimus. She had longstanding stable kidney function following transplantation with a baseline GFR of 84 mL/min and a creatinine of 80 micromol/L. Her past medical history was remarkable for iatrogenic hypothyroidism following parathyroidectomy for primary hyperparathyroidism, and she had no prior history of malignancy.

In April 2015, after ten years of immunosupression, she developed an irregular hyperpigmented and evolving lesion on her left upper back which was resected in May 2015. The biopsy of the left scapular site revealed a superficial spreading invasive melanoma with a maximum Breslow thickness of 2.59 mm, Clark level of IV, with, mitoses of 5/mm^2^. No ulceration was identified, but tumor infiltrating lymphocytes and tumor regression were present. Peripheral margins were uninvolved, but deep margin was involved by a satellite nodule and microsatellitosis was also present. Wide-local excision and bilateral axillary lymph node biopsies were performed July 2015, as lymphoscintigraphy had identified drainage of each of the lesion to the contralateral axilla when injected with technetium 99 sulfur colloid. Three right axillary lymph nodes were removed and were negative for malignancy, but 1 left axillary lymph node was removed and pathology revealed an 8.5 mm × 7.5 mm melanoma deposit with extranodal extension. As a result, the patient required a wide local excision with a 2 cm margin and pathology demonstrated residual disease and microsatellites, with negative margins. A second lesion was excised at the time of surgery in the right scapula, again consistent with superficial spreading invasive melanoma, Breslow thickness 1 mm, Clark level II, without ulceration, no mitoses and clear margins (pT1a). Completion lymph-node dissection August 2015 retrieved 22 lymph nodes, all of which were negative for melanoma, with final American Joint Committee on Cancer (AJCC) pathological staging of pT3aN2c.

Staging CT in July 2015 showed a non-specific 6.5 mm non-calcified right lower lobe (RLL) lung nodule, not previously present. *BRAF* mutation test of the with real-time PCR assay using the Qiagen *BRAF* RGQ kit was *BRAF* wild-type. The patient was not offered adjuvant radiotherapy and declined high-dose adjuvant interferon.

Follow-up CT imaging in October 2015 demonstrated increase in size of the RLL lung nodule and the appearance of at least eight new subcentimeter bilateral pulmonary nodules, along with increased mediastinal and left hilar lymphadenopathy (12 mm). The patient was asymptomatic. A follow-up 2-deoxy-2[F-18] fluoro-D-glucose (FDG) PET-CT scan in December 2015 demonstrated an intensely hypermetabolic (SUV max 9.9) left hilar lymph node enlarging to 16 mm, along with non-FDG avid pulmonary nodules. An endobronchial ultrasound-guided biopsy of the hilar lymph node (station 11 L) demonstrated atypical cells reactive for S100/melanA, confirming metastatic melanoma. Her case was discussed at the multidisciplinary tumor board and renal transplantation team, and a recommendation for anti-PD-1 treatment was made, based on available safety data and high risk of cancer-related mortality. Full discussion with patient and her husband regarding the risks and benefits of treatment were had and the patient wished to proceed with treatment including unknown risks of allograft rejection. Immunosuppressive medications were titrated off and she was left on 10 mg of prednisone daily, with no deterioration in renal function prior to nivolumab administration.

The patient received her first treatment of nivolumab (anti-PD-1 treatment for metastatic melanoma, single intravenous dose of 324 mg) on January 7^th^, 2016. She reported no subjective toxicities within the first week of treatment, but on day 8 the patient developed lethargy, abdominal pain, nausea, vomiting and loose stools (4 times per day), malaise, anorexia and fatigue. Physical examination demonstrated signs of uremia and concurrent tenderness in the lower abdomen at the site of allograft implantation without peritoneal signs. Laboratory investigations showed a creatinine rise to 577 micromol/L without any change in electrolytes. The ultrasound Doppler of her kidney showed markedly abnormal appearance of the transplant kidney with findings suggestive of acute medical renal disease, poor perfusion and elevated resistive indices concerning for transplant dysfunction. She received a pulse of corticosteroids (methylprednisolone 500 mg IV × 1), and developed diabetic ketoacidosis requiring insulin infusion and initiation of hemodialysis. She had a second pulse of steroids with close endocrinologic monitoring and insulin sliding scale, after which prednisone was tapered down. Renal allograft function did not return and she was discharged home on hemodialysis. Nivolumab was withheld and the patient was observed.

**Fig. 1:**
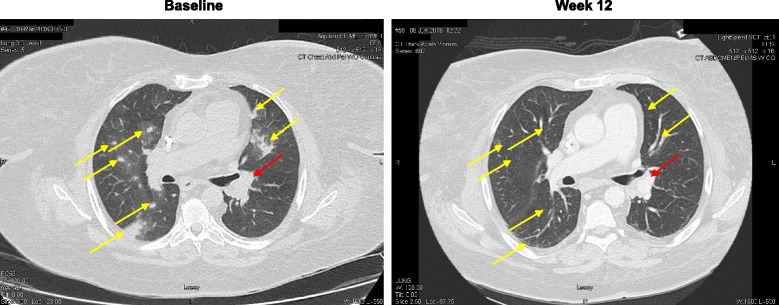
Radiographic response to nivolumab on hemodialysis; CT chest exams performed at baseline and 12 weeks after re-initiation of therapy demonstrates resolution of lung nodules (indicated by the *yellow arrows*). The hilar lymph node indicated by the *red arrow* is stable

Restaging CT thorax on February 2016 demonstrated a partial resolution of bilateral pulmonary nodules, hilar lymph nodes and mediastinal lymph nodes but right lower pleural thickening was noted. However, the patient had subsequent clinical deterioration 6 weeks later in March 2016 with dyspnea, cough and hypoxia with CT thorax showing significant progression of lung parenchymal disease and multiple new lung nodules. Infection was ruled out by bronchoscopic examination, and empiric treatment with piperacillin/tazobactam. After careful consideration and multidisciplinary review, the patient was re-administered nivolumab starting April 2016, with both ongoing clinical and radiographic response. Restaging 12-week CT thorax June 2016 on nivolumab shows almost total resolution of previously noted multiple bilateral pulmonary nodules and consolidations (Fig. 1), but some slight increase in size of mediastinal and hilar lymph nodes not meeting criteria for progression by immune-related response criteria (irRC) in solid tumors [11]. At the time of publication the patient has an ongoing (8-month) response in lung metastases and stable mediastinal/hilar lymph nodes, but slight growth of a single axillary lymph node.

## Conclusions

In this case report, we add to the expanding literature demonstrating that treatment with an anti-PD-1 antibody may be associated with transplant allograft rejection. At the time of this adverse event there had not been any previously documented cases of allograft rejection with anti-PD1 agents, and several reports of safe administration of anti-CTLA-4 [4–6]. We had originally planned for allograft extraction after the patient’s condition stabilized on hemodialysis; however, melanoma disease progression ultimately required more urgent systemic treatment and we monitored the patient closely after resumption of nivolumab. The result was an objective response to treatment to nivolumab which is ongoing. The timing of allograft rejection in reference to first-dose administration of nivolumab implies causality, especially in light of the chronicity of the allograft (>10 years) and suggests T-cell-mediated rejection [7].

We decided to stop all immunosuppression except prednisone (10 mg daily) prior to nivolumab administration. Although a more conservative reduction in immunosuppression may have prevented the transplant from failing, we were concerned of the effect of immunosuppressive medications on the anti-tumor effect of nivolumab. Moreover, a longer waiting period prior to nivolumab administration may have ruled out rejection due to titration of immunosuppressive medications, however, the patient’s aggressive course of disease suggested a faster timeline for treatment and there was the option for hemodialysis as rescue. A less aggressive reduction in immunosuppression would be warranted with other transplants (e.g. heart, liver etc.) since transplant failure would inevitably lead to patient death. The product monograph for nivolumab does not suggest an adjustment of nivolumab on hemodialysis [12–14], and in our patient this was safely and effectively administered while on hemodialysis.

This case study and similar reports [7–10] demonstrate the importance of post-marketing studies in populations excluded by pivotal and early-phase clinical trials; in this case, solid organ transplant recipients (SOTRs). Potential challenges and risks of immune-checkpoint inhibition for SOTRs include 1) strong potential for organ transplant rejection, at least with anti-PD-1/PD-L1 treatment as this case has demonstrated [4]; 2) potential for reduced anti-tumor activity of immune checkpoint inhibition, especially after titration of potent immunosuppressive medications that can reduce T-cell function; 3) complicated medical management of addressing organ failure (e.g. hemodialysis) and co-morbidities that led to organ failure (e.g. diabetes); 4) evolving treatment landscape with multiple immune checkpoints that differ in mechanism and tolerability (i.e. CTLA-4 versus PD-1) [7, 10, 15]. This case also raises the question of whether patients should be considered for the rather radical approach of allograft removal in order to become eligible for treatment with anti-PD-1 agents. Although phase III clinical studies have demonstrated that anti-PD-1 agents such as nivolumab and pembrolizumab have superior efficacy over ipilimumab in metastatic melanoma [16], individual case reports suggest that anti-CTLA-4 agents may be preferred for use in SOTRs due to the non-peripheral tissue-specific mechanism of the CTLA-4 receptor compared to PD-1, which would be less likely to be associated with acute rejection of the allograft.

It is also important to note that allograft rejection occurred with both nivolumab and pembrolizumab [7] which is expected as the two anti-PD-1 antibodies possess similar therapeutic mechanisms. We suggest that the characterization of PD-L1 expression on renal allografts prior to administering an anti-PD-1 agent may allow the stratification of patients at high risk of rejection upon treatment. To date, no studies have characterized PD-L1 expression on a series of renal transplants correlating expression levels with rejection following anti-PD-1 therapy. It has however been demonstrated that PD-1 expressing T-cell subsets are indicators of risk of rejection of renal transplants [17].

Approximately half of all SOTRs will develop a skin malignancy, with squamous cell and basal cell carcinomas accounting for 90 % [18]. SOTRs have a 2.4 increased risk of developing melanoma [19], and a retrospective review by Brewer et al. demonstrated that SOTRs with thick melanomas (Clark level III or IV or a Breslow thickness of 1.5-3.0 mm) had a significantly poorer melanoma cause-specific survival rate [20]. The introduction of effective therapies such as nivolumab in patients with prior solid organ transplants will have to take into consideration the risk of promoting foreign antibody from the graft leading to rejection, versus the benefit of treating their melanoma, especially when the prognosis is compromised. We suggest that treating SOTRs with immune checkpoint blockade could be more dangerous in non-renal transplant recipients (e.g. cardiac, pulmonary, and hepatic) where organ-replacement therapy equivalent to dialysis is unavailable [21].

The exclusion of immunosuppressed patients or patients with an existing autoimmune disorder in clinical trials investigating the safety of anti-PD-1/anti-PD-L1 agents has left us dependent on case report studies where to our knowledge, no group has been able to treat a melanoma (or other skin malignancy) with these antibodies without causing acute graft rejection [7–10]. A retrospective review of patients with advanced melanoma and pre-existing autoimmune disorders has only been conducted for patients who received ipilimumab (anti-CTLA-4) and results showed generally exacerbations of their autoimmune symptoms that were manageable [22]. Nonetheless, from murine transplant models, we have learned that the PD-1 pathway is critical for the induction and maintenance of transplantation tolerance [15]. Furthermore, CTLA-4 and PD-1 agonists are emerging as immunosuppressive agents for SOTRs [23, 24]. Therefore, graft rejection may have been predictable in this case.

While patients are now living longer and better with organ transplants, their immunosuppressive environment is allowing the development of an increasing burden of transplant-related malignancies. The development of novel immunosuppressive drugs that reduce risk of malignancy, as well as surveillance and monitoring strategies should be considered for the management of cancer in SOTRs. One emerging and promising area is the use of mTOR inhibitors for immunosuppression compared to calcineurin inhibitor-based regimens which have shown to decrease risk of malignancy [25]. Further studies to prevent and manage malignancies in this complicated situation are required.

## Abbrevations

CTLA-4: Anti-cytotoxic T-lymphocyte-associated protein-4
FDG: Fluorodeoxyglucose
GFR: Glomerular filtration rate
IgG-4: Immunoglobulin G-4
irRC: Immune-related response criteria
IV: Intravenous
PCR: Polymerase chain reaction
PD-1: Programmed death receptor-1
PD-L1: Programmed death receptor-1 ligand
PET-CT: Positron emission tomography-computed tomography
RLL: Right lower lobe
SOTR: Solid organ transplant recipient
SUV: Standardized uptake value
